# Research on the Current Development Status of Redox Flow Batteries

**DOI:** 10.3390/molecules31060943

**Published:** 2026-03-11

**Authors:** Runze Li, Han Yan, Yang Guo, Zizhen Yan, Shiling Yuan, Meng Lin

**Affiliations:** 1 Shandong Key Laboratory of Green Electricity&Hydrogen Science and Technology, School of Chemical Engineering, Shandong Institute of Petroleum and Chemical Technology, Dongying 257061, China; 2 School of Chemistry and Chemical Engineering, Shandong University, Jinan 250100, China; 3 Shida Shinghwa Advanced Material Group Co., Ltd., Dongying 257503, China

**Keywords:** redox flow batteries, electrodess, ion-exchange membraness, electrolytes, modification

## Abstract

In recent years, flow batteries have emerged as a crucial technological solution for large-scale energy storage, leveraging their unique power-capacity decoupling characteristics and long cycle life to demonstrate significant potential in applications such as renewable energy integration and grid frequency regulation. Based on differences in electrolyte systems, mainstream flow battery technologies are primarily categorized into three types: all-vanadium redox flow batteries (VRFBs), iron-chromium redox flow batteries (ICFBs), and zinc-based redox flow batteries (ZRFBs). However, each of these technologies faces critical challenges in practical commercialization: VRFBs are constrained by cost pressures due to fluctuations in vanadium resource prices and relatively low energy efficiency; ICFBs require urgent solutions to issues such as hydrogen evolution side reactions at the negative electrode and the sluggish kinetic responses of the Cr^3+^/Cr^2+^ redox couple; while ZRFBs grapple with safety concerns such as zinc dendrite growth and morphology instability. To overcome these technical bottlenecks, extensive innovative research has been conducted in key materials (electrodes, ion-exchange membranes, electrolytes). Against this backdrop, this paper systematically reviews recent advances in the modification and optimization of flow battery technologies and conducts an extended discussion on the emerging organic redox flow batteries in recent years.

## 1. Introduction

With the rapid advances in technology and industry, traditional fossil fuels face increasing skepticism and resistance due to their inherent limitations and environmental damage, while eco-friendly, efficient renewable energy sources are gaining more attention [1,2,3,4,5,6]. According to data from the International Energy Agency, despite rising global carbon dioxide emissions and accelerating energy demand, the growth of traditional fossil fuels such as oil and coal has clearly slowed. In contrast, renewable energy has experienced the most significant growth [7]. The core challenge in the development and utilization of renewable energy lies in its inherent characteristics: clean energy sources like wind and solar exhibit significant intermittency and volatility, making it difficult to directly meet the stability requirements of power grid operations [8,9,10]. Therefore, stable and controllable large-scale energy storage systems must serve as a critical support to achieve continuous, stable, and efficient energy utilization [11,12,13,14,15].

**Figure 1:**
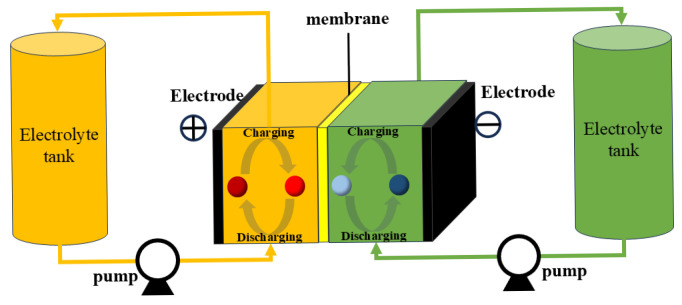
Schematic illustration of the flow battery structure.

The basic concept of redox flow battery (RFB) can be traced back to the 1930s, when Pissoort first proposed its fundamental design principles in a patent [16]. However, systematic studies of this technology were not conducted until 1976, when NASA scientists carried out comprehensive research that laid the critical foundation for the subsequent development of modern flow battery systems [17]. After 1980, sustained research by teams in Australia and Japan gradually established more engineering-feasible operational systems [18,19,20]. Through years of research, development, and practical application, RFBs have emerged as a key technology in large-scale energy storage, distinguished by their intrinsic safety, exceptionally long cycle life, flexible scalability, and excellent environmental compatibility [21,22,23,24]. The core structure of flow batteries comprises two main modules: external tanks that separately store the positive and negative electrolyte active materials, and an electrochemical stack unit composed of electrodes, an ion-exchange membrane, and bipolar plates. These components are interconnected by a pump-pipeline circulation system that enables continuous electrolyte delivery and reaction control (Figure 1). The working mechanism of RFBs is based on the reversible redox reactions of active materials on the electrode surfaces. During charging, electrical energy drives the oxidation of active materials at the positive electrode and reduction at the negative electrode, converting electrical energy into chemical energy stored in the electrolytes. During discharge, the reverse reactions occur, converting the stored chemical energy back into electrical output [25,26,27]. These technical characteristics position flow batteries not only as a critical solution for addressing the intermittency challenges of renewable energy but also as vital energy storage support for enabling the transition to clean energy, mitigating the greenhouse effect, and alleviating ecological crises [28,29,30].

## 2. Redox Flow Battery

Current mainstream RFB technologies primarily comprise three major types: all-vanadium redox flow batteries (VRFBs), iron-chromium redox flow batteries (ICFBs), and zinc-based redox flow batteries (ZRFBs) [19,31,32,33,34]. Their core differences primarily lie in the active materials involved in the electrochemical reactions within the positive and negative electrolytes. These systems exhibit distinct characteristics in terms of technical performance, cost structure, and application scenarios, with significantly divergent research and development priorities. Research on VRFBs focuses on enhancing energy efficiency (EE) and minimizing capacity loss to alleviate cost pressures associated with expensive vanadium raw materials [35,36,37]. ICFBs aim to address challenges related to sluggish reaction kinetics and side reactions of chromium ions at the negative electrode [38,39]. ZRFBs concentrate on overcoming issues of uneven zinc deposition and dendrite formation [40,41]. Current research on RFBs primarily focuses on core components such as ion-exchange membranes, electrolytes, and electrodes [42,43,44]. The following sections will elaborate on these three mainstream RFB systems in sequence, systematically reviewing recent innovative breakthroughs in modification research targeting the aforementioned core components. At the same time, a certain extended introduction to the emerging organic redox flow batteries (ORFBs) in recent years will also be provided.

### 2.1. Vanadium Redox Flow Batteries

VRFBs are electrochemical energy storage systems based on the redox reactions of vanadium ions (V^2+^/V^3+^ and VO^2+^/VO_2_^+^) [45,46]. The electrochemical reaction mechanism of VRFBs is as follows:

Positive electrode reaction: VO^2+^ + H_2_O − e^−^ ⇌ VO_2_^+^ + 2H^+^;

Negative electrode reaction: V^3+^ + e^−^ ⇌ V^2+^;

Overall reaction: VO^2+^ + H_2_O + V^3+^ ⇌ VO_2_^+^ + V^2+^ + 2H^+^.

The initial concept was proposed in 1984 by the team of Skyllas-Kazacos. This system first adopted a single-vanadium electrolyte design, fundamentally solving the cross-contamination issue caused by different active materials in traditional flow batteries, thereby laying the theoretical foundation for the subsequent development of VRFBs [47]. By the 1990s, Japan’s Sumitomo Electric Industries collaborated with Kazacos’ team to advance VRFB technology from laboratory research to engineering exploration. They successfully developed the first kilowatt-scale demonstration system, marking a critical step toward practical application [48,49]. Post-2010, VRFBs gradually became one of the mainstream technologies for large-scale energy storage. Countries and regions, including China, the United States, and Japan, deployed multiple megawatt-scale projects, with a representative example being the 200 MW/800 MWh Dalian, China project, which fully validated the engineering feasibility of grid-level energy storage [50,51,52].

The U.S. Department of Energy has set a target cost reduction goal of $150/kWh for fully integrated distributed energy storage systems [53]. However, due to the persistent rise in the price of the raw material vanadium, it once soared to $75,000 per ton [54,55]. The operating costs of VRFBs have risen accordingly, with electrolyte costs alone climbing to approximately $122/kWh in recent years [56]. Therefore, the research focus of VRFBs has gradually shifted toward improving overall battery EE, reducing the rate of capacity degradation during operation, and enhancing vanadium utilization efficiency over the full lifecycle. These efforts aim to reduce the total system cost through technological advancements and strengthen its competitiveness in the large-scale energy storage market.

#### 2.1.1. Ion-Exchange Membranes

Research on membrane modification primarily focuses on three core performance metrics: conductivity, ion selectivity, and durability [57,58,59]. Enhancing conductivity effectively reduces interfacial resistance within the battery and improves mass transfer efficiency, thereby lowering energy loss and increasing EE [60]. Ion selectivity directly influences the crossover behavior between the positive and negative electrolytes—during battery operation, vanadium ions migrate across the ion-exchange membrane, leading to electrolyte concentration imbalance, reduced Coulombic efficiency (CE), accelerated capacity decay, and shortened battery life [61]. Given that VRFBs operate in a strongly acidic electrolyte environment, improving the membrane’s corrosion resistance is a critical measure for ensuring long-term operational stability [62].

In commercial VRFBs, perfluorosulfonic acid membranes like Nafion are predominantly used. These membranes offer high conductivity and excellent chemical stability. They provide efficient ion transport channels and ensure long-term operational reliability for the battery [63,64]. However, these membrane materials face significant technical challenges. On one hand, their microscopic channel structure shows poor selectivity toward vanadium ions. This leads to severe vanadium ion crossover. As a result, it reduces the battery’s CE and causes electrolyte concentration imbalance and capacity decay [65,66]. On the other hand, the complex synthesis process of perfluorosulfonic acid resin and the requirement for high-purity raw materials make their manufacturing cost high (responsible for about 11 % of the overall cost of a 1 MW/8 MWh system) [67]. These factors collectively limit the widespread application of VRFBs in broader scenarios.

To overcome these limitations, the research focus has gradually shifted to new membrane material systems. Some non-fluorinated polymer matrix, such as sulfonated poly(ether ether ketone) (SPEEK) or polybenzimidazole (PBI), offers lower raw material costs and better environmental compatibility [68,69,70,71]. However, they also have drawbacks, including poor conductivity and low stability. Therefore, incorporating nanomaterials into the polymer matrix to prepare mixed-matrix membranes has become a key research focus [72]. Wu applied an asymmetric hydroxyphosphorylation functionalization strategy to modify an imine-linked covalent organic framework (COF) through post-synthetic modification. The phosphorated covalent organic framework nanoparticles prepared via this process can be embedded within a PBI matrix to construct a hybrid matrix membrane. The resulting membrane achieved a significant increase in proton conductivity, from 0.031 to 0.058 S cm^−1^, along with enhanced chemical stability. When used in VRFBs, the membrane demonstrated excellent performance, reaching an EE of 85.5% at 120 mA cm^−2^ [73]. Shahi grafted defluorinated PVDF-co-HFP with the monomer [2-(methacryloyloxy)ethyl]dimethyl-(3-sulfopropyl)ammonium hydroxide. Subsequently, the grafted polymer was doped with 3.0 wt% of sulfonated functionalized graphene oxide. This composite membrane demonstrated ultra-high ion selectivity in VRFB tests, measuring 1.85 × 10^5^ S min cm^−3^. At a current density of 100 mA cm^−2^, the battery achieved an EE of 84.21%. The membrane also showed no performance degradation over 350 consecutive charge–discharge cycles. This study confirms that the synergistic effect between functionalized nanofillers and zwitterionic polymers is an effective strategy for developing high-performance ion-selective membranes [74]. Moreover, Kaur dispersed tin dioxide nanoparticles within a SPEEK matrix, thereby preparing a series of tin dioxide/SPEEK composite ion exchange membranes. The membrane containing 2 wt% nanoparticles demonstrated excellent overall performance. It exhibited a tensile strength of 32.48 MPa and a low vanadium ion permeability of 4.22 × 10^−9^ cm^2^ s^−1^. When tested in a VRFB, the membrane achieved an EE of 86.47% and maintained stable operation for 100 cycles [75]. Zhang incorporated the modified metal–organic framework (MOF)into the SPEEK matrix. The resulting composite membrane exhibited an ultrahigh ion selectivity of 192.7 × 10^3^ S·min·cm^−3^, which is 49 times greater than that of a pristine SPEEK membrane. When applied in a VRFB operating at a current density of 100 mA·cm^−2^, the battery achieved an EE of 87.1%. Moreover, the composite membrane showed significantly enhanced mechanical and chemical stability. Its self-discharge time extended to hundreds of hours, and it maintained excellent performance stability over 300 charge–discharge cycles [76].

In recent years, research on ion-exchange membranes for VRFBs has focused on mixed matrix membranes. The matrix employs non-fluorinated polymers, which inherently offer substantial cost advantages. Functionalized nanofillers are incorporated to synergistically enhance proton conductivity, vanadium ion selectivity, and chemical stability. This strategy effectively suppresses vanadium-ion crossover while preserving the polymer matrix’s low cost. Experimental results confirm that properly engineered non-fluorinated membranes can surpass conventional Nafion membranes in both EE and cycle life (Table 1). Future trends will focus on the development of low-cost, highly environmentally compatible membrane materials. These will integrate polymeric nanomaterials with green synthesis processes to promote scaled-up production, substantially reduce the operational costs of VRFBs, and simultaneously enhance battery efficiency.

#### 2.1.2. Electrolytes

The electrolyte in vanadium VRFBs is an aqueous solution of multivalent vanadium ions and sulfuric acid. Research on electrolyte modification focuses on improving the solubility and stability of vanadium electrolytes [77]. The core challenge stems from the demand for high energy density in VRFBs. To achieve this goal, the vanadium concentration usually needs to be maintained in the range of 1.5 M to 1.8 M. However, when the vanadium concentration exceeds 1.5 M, the electrolyte system is in a high ionic strength state. Under fluctuating operating temperatures (10–40 °C), this can easily lead to polymerization and precipitation of vanadium ions. To suppress precipitation formation, the sulfuric acid concentration needs to be precisely controlled at around 3 M. This provides a sufficiently acidic environment to maintain vanadium ion dissolution. Under these conditions, the vanadium ions in the electrolyte are actually in a quasi-supersaturated or metastable state, posing a risk of spontaneous precipitation [78]. Therefore, the key objective of electrolyte modification is to regulate its thermodynamic stability and kinetic dissolution behavior. This ensures effective dissolution of vanadium ions over a wide temperature range, thereby guaranteeing long-term stable operation of the battery.

Wei investigated the effects of Ti^4+^, Fe^3+^ and Cr^3+^ ionic impurities on the electrolyte, with these impurities primarily originating from vanadium-titanium magnetite-based raw materials. The findings indicated that Ti^4+^ ions in the electrolyte can effectively form stable complexes with VO_2_^+^ ions. This interaction not only prevents VO_2_^+^ precipitation but also enhances the thermal stability of the electrolyte. When the Ti^4+^ concentration reached 0.02 mol L^−1^, it most significantly improved the electrochemical activity of the VO^2+^/VO_2_^+^ couple. The peak current increased by 9.66% and 11.51%, respectively. The ion diffusion coefficient also increased by 19.8% and 17.6%. Furthermore, the addition of Ti^4+^ effectively mitigated the negative effects caused by Fe^3+^ and Cr^3+^ impurities. In electrolytes containing Fe^3+^/Cr^3+^ impurities, the addition of Ti^4+^ significantly improved both voltage efficiency (VE) and EE compared to electrolytes without Ti^4+^. Furthermore, the cycling stability of the VRFB was greatly enhanced, retaining a capacity of 146 mAh after 100 cycles [79].

Zhao synthesized a novel aqueous ionic liquid-based electrolyte for VRFBs. They employed a strategy using 1-butyl-3-methylimidazolium chloride ionic liquid and vanadium chloride. This electrolyte significantly increased the solubility of the vanadium salt, with a maximum achievable concentration of about 2 mol L^−1^. It also enhanced the overall battery performance. At room temperature, the electrolyte exhibited a dynamic viscosity of 36.62 mPa·s, an ionic conductivity of 0.201 S cm^−1^, a stable potential window of approximately 1.8 V, and the theoretical energy density reached 44.24 Wh L^−1^. VRFBs using this electrolyte achieved a CE and capacity retention both exceeding 85% at a discharge current of 5 mA [80].

Zarei-Jelyani fabricated an optimized nanofluid electrolyte by precisely controlling multi-walled carbon nanotube (MWCNT) concentration within the range of 0.004–0.020 wt%. This was combined with ultrasonic treatment and high-speed homogenization techniques. The nanofluid with 0.012 wt% MWCNTs demonstrated the best performance. In a three-electrode system, it achieved a 43% increase in peak current, a 20–22% reduction in polarization, and a 72% enhancement in diffusion kinetics [81].

Research on electrolyte modification for VRFBs focuses on resolving the core challenge of vanadium ion instability and precipitation at high concentrations. Recent strategies have expanded to include: incorporating metal ions to form stable coordination complexes; developing novel ionic liquid electrolytes; and introducing nanofluids. These approaches synergistically enhance electrolyte performance through thermodynamic regulation and kinetic optimization. Future improvements to VRFB electrolytes may focus on deepening the integration of established pathways, synergistically driving engineering breakthroughs characterized by “high concentration, wide temperature range, and low cost”. This will provide core support for the large-scale application of VRFB in the energy storage sector.

#### 2.1.3. Electrode Materials

The electrodes of conventional VRFBs primarily utilize porous carbon-based materials such as carbon felt (CF) and graphite felt (GF). These materials, characterized by their three-dimensional porous structure, are considered ideal electrode substrates in VRFB systems due to their excellent electrical conductivity, good structural stability, and low cost [82]. In recent years, emerging carbon-based materials like carbon paper (CP) and carbon cloth (CC) have also been explored and applied [83,84,85]. In practice, these electrode materials usually undergo pretreatment processes, such as heat treatment or acid treatment. This increases the surface oxygen-containing functional groups, enhances hydrophilicity, and thereby improves their wettability with the electrolyte [86,87]. However, such materials still possess inherent drawbacks, including insufficient intrinsic electrocatalytic activity and a low overpotential for the hydrogen evolution reaction (HER). These limitations constrain further improvements in battery electrochemical efficiency [88,89,90,91]. To address this limitation, current electrode modification research focuses on methods such as doping with metal/metal oxide nanoparticles or introducing non-metallic materials with high catalytic activity. These approaches aim to enhance the electrocatalytic activity of the electrode surface and suppress the HER side reaction. Consequently, this accelerates the kinetics of the vanadium ion redox reactions, ultimately achieving a synergistic improvement in both the EE and cycling stability of the battery [92,93,94,95].

To enhance the energy efficiency and long-term cycling stability of VRFBs, significant research efforts have been directed toward advanced electrode design and functionalization. These strategies aim to simultaneously improve electrochemical kinetics, facilitate mass transport, and suppress detrimental side reactions such as the hydrogen evolution reaction (HER). Recent progress can be categorized into several interconnected approaches: constructing heterogeneous composite structures, engineering micro-/nanoscale architectures, and achieving atomic-level synergistic modulation. A prominent strategy involves integrating conductive scaffolds with highly active catalytic phases. For instance, a uniformly dense MXene–SnO_2_ composite layer on carbon felt leverages the high electrical conductivity of MXene and the abundant catalytic sites of SnO_2_. The synergistic interaction between these components significantly enhances electrochemical activity, enabling an EE of 85% at 100 mA cm^−2^ and a discharge capacity of 2464.69 mAh, alongside exceptional stability over 500 charge–discharge cycles with no performance decay [96].

Beyond material composition, precise control over electrode microstructure has proven highly effective. The fabrication of nitrogen-doped vertically oriented graphene on graphite felt via a metal-free chemical vapor deposition method creates vertically aligned channels that eliminate mass transport limitations. Concurrently, nitrogen functionalization enhances vanadium redox kinetics through strong ion adsorption, as supported by DFT calculations. This rationally designed electrode achieved an EE of 87.1% at 200 mA cm^−2^ and maintained an efficiency above 80.2% after 1500 cycles at 300 mA cm^−2^, while also attaining a peak power density of 1308.56 mW cm^−2^. Notably, this metal-free process inherently avoids HER catalyzed by metallic residues [97]. Pushing the frontier to atomic-scale engineering, the construction of specific coordination bonds on electrodes has emerged as a powerful route to tailor electronic structures and reaction pathways. A representative example is a bismuth–nitrogen/carbon co-functionalized graphite felt electrode, where Bi–N and Bi–C coordination bonds were formed. These bonds synergistically modulate the local electronic environment, facilitating vanadium ion adsorption and structurally anchoring active sites, thereby accelerating V^2+^/V^3+^ kinetics. Furthermore, this configuration positively shifts the hydrogen evolution onset potential, effectively suppressing HER. Consequently, the VRFB equipped with this electrode delivered an EE of 80.26% at 220 mA cm^−2^, a peak power density of 780.3 mW cm^−2^, and outstanding cycling stability with 98.2% efficiency retention after 600 cycles and minimal capacity fade [98]. In summary, the evolution of VRFB electrode design is progressing from simple surface modification toward the sophisticated integration of composite engineering, morphological control, and atomic-level precision. The overarching goal is to create multifunctional electrodes that concurrently address the interconnected challenges of slow kinetics, inefficient mass transport, and parasitic reactions. Future development will likely rely on deeper mechanistic insights and the rational design of tailored interfaces to further unlock the performance ceiling of VRFBs for large-scale energy storage.

Recently, VRFB electrode modification research has explored optimal materials through innovative approaches that combine carbon-based electrodes with various dopants. These breakthroughs have significantly enhanced electrochemical catalytic activity and stability, with EE improvements of 5–20%. Modified materials stably bond to the matrix, maintaining degradation rates below 5% after 500–1500 cycles (Table 2). The exploration of VRFB electrode materials indicates further leaps in battery performance. Future research will focus on achieving high performance, long lifespan, low cost, and environmental friendliness through interdisciplinary integration, aiming to overcome current technical barriers and provide reliable, efficient solutions for large-scale energy storage applications.

### 2.2. Iron-Chromium Redox Flow Batteries

ICFBs are one of the earliest developed flow battery systems. Its foundational research and practical exploration were jointly advanced by NASA in the United States and Japan’s Mitsui during the 1970s–1980s [99]. The electrochemical reaction mechanism of ICFBs is based on the redox reactions of iron/chromium ions:

Positive electrode reaction: Fe^2+^ − e^−^ ⇌ Fe^3+^;

Negative electrode reaction: Cr^3+^ + e^−^ ⇌ Cr^2+^;

Overall reaction: Fe^2+^ + Cr^3+^ ⇌ Fe^3+^ + Cr^2+^.

Owing to the limited redox potential difference in the Fe/Cr electrode pair, ICFB typically exhibits lower energy density than VRFB. However, its core advantage lies in significantly reduced theoretical costs—iron and chromium reserves in the Earth’s crust far exceed those of vanadium, ensuring ample raw material supply and stable pricing. Although vanadium flow batteries currently enjoy greater commercialisation, persistently high vanadium prices position iron-chromium flow batteries as a compelling alternative in large-scale energy storage applications due to their cost competitiveness (the cost of electrolytes is only $17/kWh) [38,56]. To date, megawatt-scale ICFB demonstration projects have been deployed in several countries, including the United States and China, thereby validating their engineering feasibility [43,100].

Nevertheless, a major challenge remains in ICFB development, for example, the sluggish reaction kinetics at the negative electrode, particularly the slow reduction rate of Cr^3+^. The sluggish reaction kinetics at the negative electrode restrict the electrochemical reaction rate. This limitation leads to a noticeable loss inVE. Furthermore, the process is also accompanied by side reactions, such as the HER. The combined effect of these factors severely constrains the power density output of the battery. Consequently, current research focuses on enhancing the reaction kinetics through approaches such as electrode modification, electrolyte optimization, and ion-exchange membrane adjustment, aiming to drive practical application breakthroughs in ICFB technology [101,102,103,104].

#### 2.2.1. Ion-Exchange Membranes

ICFBs operate at elevated temperatures, as the high-temperature conditions prove crucial for effectively suppressing chromium aging and precipitation, thereby enhancing the long-term stability of the cell system [105,106]. However, this operating environment poses significant challenges to the performance of ion-exchange membranes. Elevated temperatures accelerate thermal oxidative degradation and chemical structural damage to the membrane material, substantially shortening the service life of the exchange membrane. High temperatures also diminish the ion selectivity of the membrane, significantly increasing the risk of iron/chromium ion cross-migration. This ultimately exerts a detrimental effect on both the CE and energy density of the batteries [107].

Among existing membrane materials, Nafion membranes are regarded as the optimal choice for ion-exchange membrane fuel cells (ICFBs) due to their comprehensive performance characteristics. This membrane exhibits outstanding chemical stability and exceptionally high proton conductivity, with its high-temperature tolerance particularly well-suited to the operational requirements of ICFBs. Although Nafion membranes carry a relatively high cost per unit area, the low cost of raw materials such as iron and chromium in ICFBs keeps the proportion of membrane material within the overall cell cost relatively manageable. Consequently, Nafion membranes remain the preferred membrane material for ICFB applications [108,109,110].

In recent years, the pursuit of cost reduction has driven innovative research into alternative membranes for ion-exchange membranes. For instance, Qiao developed a low-cost composite membrane by impregnating Nafion resin into a porous polyethylene scaffold using dimethylacetamide as the optimized solvent. The confinement effect of the porous scaffold effectively suppresses Nafion swelling and stabilizes ion transport channels. Meanwhile, sulfonic acid groups in the Nafion resin formed a continuous proton-conducting network. Experimental results showed that the membrane exhibits significantly lower water swelling than conventional Nafion membranes. It achieved a mechanical strength of 14.79 MPa and an ion selectivity 2.34 times higher than Nafion 115. Under operating conditions of 65 °C and 80 mA cm^−2^, ICFBs employing this membrane delivered a CE of 93.29% and an EE of 75.39%. Performance remained stable after 300 charge–discharge cycles [111].

#### 2.2.2. Electrolytes

ICFBs commonly employ a mixed electrolyte strategy. In this approach, the positive and negative electrolytes are pre-mixed before use. This design aims to balance the electrolyte composition at the source, effectively mitigating iron and chromium ion crossover caused by the imperfect selectivity of the membrane [101,112]. According to the previous research, the battery exhibits optimal performance when the mixed electrolyte composition reaches 1 M FeCl_2_ + 1.3 M CrCl_3_ + 3 M HCl [103].

Current research into modifying mixed electrolytes primarily follows two approaches. The first involves introducing catalytic metal ion additives, such as Bi^3+^ and In^3+^. Upon adsorption onto the electrode surface, these ions exert a catalytic effect, significantly enhancing the reaction kinetics of the Cr^3+^/Cr^2+^ and Fe^2+^/Fe^3+^ redox couples [113,114,115]. For example, Ye investigated the impact of trace metal species (Cu^2+^, Ni^2+^, Bi^3+^) on HER at CF in hydrochloric acid-based flow batteries. Through three-electrode electrochemical tests and in situ polarization in 3 M HCl electrolytes, they revealed distinctly different mechanisms of action for the components. An ultra-low Cu loading (12.5 μg cm^−2^) reduced HER overpotential by 100 mV; soluble Ni^2+^, at concentrations exceeding 200 μM, acted as an endogenous catalyst, shifting the negative electrode potential positively by approximately 100 mV; Bi^3+^ alone exhibited minimal promotion of the HER, but in co-existence with Cu/Ni, it effectively suppressed Cu/Ni-induced HER, shifting the electrode potential negatively by up to 50 mV. These findings provide crucial guidance for controlling the purity of industrial electrolytes and reveal the strategic application of the dual-function additive bismuth. By enhancing primary redox kinetics while suppressing parasitic HER, it significantly improves the reliability and lifespan of iron-chromium flow batteries for grid-scale energy storage [116]. Niu introduced PbCl_2_ as an additive into ICFBs electrolytes to achieve in situ electrodeposition of lead-based catalysts on carbon electrodes. Molecular dynamics simulations and electrochemical analyses revealed that 40 mM Pb^2+^ formed catalytic Pb and Pb(ClO_3_)_2_ deposits on the electrode surface during cycling, which significantly enhanced Cr^3+^/Cr^2+^ reaction kinetics while suppressing HER. At 140 mA cm^−2^, the modified system delivered 83.90% EE (5.68 percentage points higher than the baseline) with 97.4% CE and 86.1% VE. In situ differential electrochemical mass spectrometry confirmed a substantial reduction in hydrogen evolution signals. The battery operated stably for over 400 cycles with minimal capacity decay. The above simple, low-cost electrolyte additive strategies eliminate complex electrode preparation procedures while simultaneously addressing sluggish chromium kinetics and HER challenges in ICRFBs [117].

The second approach involves adding specific chelating agents. These molecules form stable chelate complexes with iron and chromium ions in solution, effectively inhibiting the hydrolysis reactions of metal ions. This method not only reduces the extent of metal ion hydrolysis side reactions but also enhances the apparent solubility and hydrogen electrode potential by altering the ionic species present, improving the electrochemical efficiency of the battery [118,119,120,121]. Marshak isolated and structurally characterized a highly reduced aqueous chromium(II) complex (Cr-PDTA), which operated stably at −1.10 V vs. SHE (pH = 9) without HER. This study elucidated the mechanisms governing the Cr^2+^/Cr^3+^ redox pair in aqueous solutions at the molecular level. It demonstrated that kinetic barriers, rather than thermodynamic constraints, dominate the actual stability in aqueous systems [122]. Li introduced guanidine hydrochloride as an additive to modulate the solvation structure of chromium ions in electrolytes. Molecular dynamics simulations and ultraviolet-visible spectroscopy analysis indicated that the additive enhances redox kinetics by promoting electron transfer through increasing the coordination number of chloride ions in the first hydration layer of Cr ions. In situ Raman spectroscopy confirmed that the additive disrupts the hydrogen-bonded water network near the electrode surface, thereby inhibiting the HER by impeding proton transport. At the optimal concentration, the modified electrolyte achieved 80% EE at 55 °C, with significantly reduced hydrogen evolution and 46.0% remaining capacity after 20 cycles [123]. Ai developed a neutral ICFB employing a symmetric chelation strategy with 1,3-diaminopropane tetraacetic acid as the identical ligand for both Fe^3+^/Fe^2+^ and Cr^3+^/Cr^2+^ complexes. FTIR and UV-vis spectroscopy confirmed the formation of stable coordination compounds with high solubility in neutral aqueous solutions. This symmetric ligand design effectively suppressed crossover of active species while enhancing redox kinetics of the sluggish Cr^3+^/Cr^2+^ couple. The resulting ICFBs demonstrated exceptional performance metrics, including a discharge energy density of 12.1 Wh/L, an average capacity decay rate of merely 0.13% per cycle over 300 cycles, and nearly 100% CE at 50 mA/cm^2^ with 60% EE. By eliminating corrosive acids from traditional electrolytes, this approach significantly reduced material degradation and operational risks while maintaining performance comparable to conventional acidic systems [124].

Electrolyte modification in ICFBs has achieved significant breakthroughs in traditional acidic systems by synergistically regulating catalytic metal ions and designing symmetrical chelating agents. Future research will focus on three directions: First, deepening the “catalysis-suppression” synergy to develop multifunctional additive systems that precisely match Cr^3+^/Cr^2+^ kinetic enhancement with HER suppression. Second, promoting acid-free chelated electrolyte systems through molecular structure optimization to eliminate corrosive acids, improving system safety and material longevity. Third, exploring novel electrolyte systems to reduce operating temperatures and costs. These strategies will collectively advance ICFBs toward engineering applications with high energy density, long cycle life, and full-condition stability, providing economically viable solutions for large-scale grid energy storage.

#### 2.2.3. Electrode Materials

The functionalized electrodes in ICFBs, similar to those in VRFBs, are also predominantly carbon-based materials (e.g., CF, GF, CP, CC), sharing the same advantages mentioned previously [125,126,127]. Regarding electrode modification, the Fe^2+^/Fe^3+^ redox couple in ICFBs cathodes exhibits inherently high redox activity, typically allowing direct use after simple pretreatment [128]. Some studies continue to explore the incorporation of active catalysts to enhance the electrochemical kinetic performance. For example, Niu fabricated an N-B co-doped TiB_2_/CC electrode via a simple impregnation-carbonization method for use as two electrodes in ICFBs. The modified electrode exhibited significantly enhanced specific surface area and hydrophilicity. The synergistic effect of N-B doping and Ti catalysis accelerated the reaction kinetics of both Cr^3+^/Cr^2+^ and Fe^3+^/Fe^2+^ redox couples. After 50 cycles at 140 mA cm^−2^, the discharge capacity increased by 72.2%, and the EE reached 82.7% [129].

The key research challenges and core difficulties for ICFBs are concentrated on the negative electrode side. The Cr^3+^/Cr^2+^ redox couple suffers from sluggish reaction kinetics and exhibits poor reversibility, which constitutes a critical bottleneck limiting overall battery performance [130,131]. Recent research has revealed that bismuth-based materials exhibit a unique multifunctional catalytic effect in the negative electrodes. The active materials not only effectively catalyze the reduction reaction of Cr^3+^, significantly improving the reaction rate and reversibility, but also competitively suppress the HER, a major side reaction, by altering the electrode interface properties [132,133,134]. Owing to its excellent properties, bismuth has become a research focus for the negative electrode modification. For instance, bismuth has been combined with other materials to prepare composite modified electrodes [135,136,137,138,139] or Tin, as a homologous element to bismuth, as a homolog of bismuth, has also emerged as a research focus in recent years [140].

Advancing electrode design is equally critical for enhancing the performance of ICFBs, where a primary challenge lies in accelerating the sluggish Cr^3+^/Cr^2+^ redox kinetics while concurrently inhibiting the competing HER. One promising avenue involves leveraging the cocktail effect of high-entropy alloys. For instance, a surfactant-assisted sintering process was used to fabricate a BiInSnFeTi-modified graphite felt. The cooperative interaction among these five metallic components creates synergistic catalytic sites that not only enhance charge transfer for chromium reactions but also effectively suppress HER. This balanced activity resulted in a stable EE of 75% at 120 mA cm^−2^ over 400 cycles, with a remarkably low capacity decay rate of 0.26% per cycle [141]. Concurrently, constructing hierarchical architectures from designed precursors has shown great efficacy. A multi-dimensional Bi@C electrocatalyst, derived from a bismuth-based metal–organic framework, integrates zero-dimensional carbon-coated Bi nanospheres within a two/three-dimensional porous carbon network. This structure synergistically facilitates rapid electron/ion transport and exposes abundant active sites, enabling the battery to achieve 86.08% EE at 80 mA cm^−2^ [142]. Alternatively, scalable electrodeposition of Sn nanoparticles onto graphite felt offers a more direct route. The Sn nanoparticles mediate chloride-bridged coordination with chromium species, stabilizing key intermediates and raising the energy barrier for hydrogen adsorption. This dual-function electrode delivered 79.39% EE at a high current density of 200 mA cm^−2^, with its industrial viability further validated by stable operation in a scaled 1800 W stack over 100 cycles [143].

At the atomic level, engineering heterointerfaces presents a powerful strategy for electronic structure modulation. A Sn/SnO_x_ heterojunction electrode, synthesized from a tin-terephthalate framework precursor, creates synergistic sites where metallic Sn promotes Cr^3+^ adsorption and SnO_x_ facilitates electron transfer. This interface optimization, corroborated by DFT calculations which revealed increased orbital occupancy near the Fermi level, enabled an EE of 80.5% at 160 mA cm^−2^ with stable cycling [144]. In summary, the progression in ICFB electrode design underscores a paradigm shift toward multifunctional engineering. The integration of multi-component synergy, hierarchically porous conduction pathways, and precisely constructed heterointerfaces collectively addresses the intrinsic kinetic and selectivity bottlenecks. Future development will likely focus on deepening the mechanistic understanding of these synergistic effects and exploring novel composite systems to unlock the full potential of ICFBs for cost-effective, long-duration energy storage.

Electrode modification research in ICFBs has focused on overcoming the kinetic bottleneck of the Cr^3+^/Cr^2+^ redox couple at the negative electrode. Strategies such as Bi/Sn-based synergistic catalysis, high-entropy alloy electrodes, and interface engineering have significantly enhanced reaction kinetics while suppressing HER. Combined with Table 3, it can be observed that ICFBs still lag behind VRFBs in overall EE and cycle life. However, the performance of the modified batteries has basically improved from around 60% to 70% to over 80%, and their cycle stability within 100 cycles is also quite excellent. Future electrode modification for ICFBs will continue to target the negative electrode challenges: firstly, expanding Bi/Sn-based composite catalyst systems to simultaneously strengthen Cr^3+^/Cr^2+^ kinetics and suppress HER; secondly, advancing green, low-energy fabrication processes compatible with wide-temperature operation, ensuring cost control and stability to improve system safety and reduce operational costs; thirdly, exploring novel catalysts to break through current performance limitations. These directions will collectively drive the iterative development of modified electrodes toward “high activity, long lifespan, and scalable production”, providing core support for the economic viability and reliability of ICFBs in large-scale energy storage applications.

### 2.3. Zinc-Based Redox Flow Batteries

Zinc is abundant and relatively inexpensive to extract. As one of the most reactive metals, Zinc undergoes stable electrodeposition from aqueous electrolytes [145]. The cost of VRFB electrolytes continues to rise due to increasing vanadium raw material prices. While ICFBs feature low-cost active materials, their requirement for high-temperature operation further elevates operational expenses. ZRFBs, benefiting from abundant zinc reserves and low material costs, achieve electrolyte costs as low as $5/kWh and operate at ambient temperatures. Therefore, ZRFBs have been a significant research focus in energy storage technology since the 1970s energy crisis [41,146,147,148]. After decades of development, various ZRFB systems have emerged. These systems are categorized based on the soluble redox couples used at the positive electrode. The more mature systems include zinc-bromine (Zn-Br_2_), zinc-iodine (Zn-I_2_), zinc-iron (Zn-Fe), zinc-manganese (Zn-Mn), and zinc-sulfur (Zn-S). In all these systems, zinc serves as the negative active substance, undergoing the Zn^2+^/Zn deposition/dissolution reaction. The positive electrode employs corresponding redox-active species, such as Br_2_/Br^−^, I_2_/I^−^, and MnO_2_/Mn^2+^ [149,150,151,152,153]. Among these systems, Zn-Br_2_ and Zn-Fe batteries have reached a higher technology readiness level. They have progressed to the demonstration project stage, whilst most other ZRFB battery systems remain primarily at the laboratory research and development phase [40,151].

The electrochemical reactions for key representative ZRFB systems are listed below.

Negative Electrode Reaction: Zn^2+^ + 2e^−^ ⇌ Zn.

Zinc-Bromine Flow Battery (Zn-Br_2_)

Positive Electrode Reaction: 2Br^−^ − 2e^−^ ⇌ Br_2_;

Overall Reaction: Zn^2+^ + 2Br^−^ ⇌ Zn + Br_2_.

Zinc-Iodine Flow Battery (Zn-I_2_)

Positive Electrode Reaction: 2I^−^ − 2e^−^ ⇌ I_2_;

Overall Reaction: Zn^2+^ + 2I^−^ ⇌ Zn + I_2_.

Zinc-Iodine Flow Battery (Zn-Fe)

Positive Electrode Reaction: Fe^2+^ − e^−^ ⇌ Fe^3+^;

Overall Reaction: Zn^2+^ + 2Fe^2+^⇌ Zn + 2Fe^3+^.

Zinc-Manganese Flow Battery (Zn-Mn)

Positive Electrode Reaction: Mn^2+^ + 2H_2_O − 2e^−^ ⇌ MnO_2_ + 4H^+^;

Overall Reaction: Zn^2+^ + Mn^2+^ + 2H_2_O ⇌ Zn + MnO_2_ + 4H^+^.

Zinc-Iodine Flow Battery (Zn-S)

Positive Electrode Reaction: S − 2e^−^ ⇌ S^2+^;

Overall Reaction: Zn^2+^ + S ⇌ Zn + S^2+^.

Despite the promising potential, ZRFBs face significant challenges, particularly the critical issue of zinc dendrite growth. During battery cycling, zinc forms needle-like or dendritic structures (dendritic crystals) rather than a flat, dense metallic layer. This uneven deposition reduces the utilization of the active material. More severely, growing dendrites can puncture the membrane separator. This leads to cross-contamination of electrolytes and potentially causes internal short circuits. These phenomena severely compromise battery cycle life and safety [154,155,156]. Additionally, each ZRFB system contends with its own specific side reactions. For instance, in Zn-Br_2_ batteries, the formation of insoluble zinc bromide complex precipitates, or the evolution of Br_2_ at elevated temperatures, significantly impairs CE and cycling stability [157]; in Zn-I_2_ batteries, I_2_ readily coordinates with I^−^ to form I_3_^−^, leading to the loss of active material and capacity fade [158]; in Zn-Fe batteries, iron ions are prone to hydrolysis, generating precipitates that not only deplete active species but also block electrode pores and increase interfacial impedance [159]; in Zn-Mn batteries, zinc and manganese ions tend to form inert hetaerolite, resulting in irreversible capacity loss [160], and the precipitation of zinc sulfide in Zn-S batteries similarly impedes the efficient progression of electrode reactions [161]. Therefore, the primary scientific research direction for ZRFBs is focused on effectively suppressing zinc dendrite growth and mitigating various side reactions. Overcoming these performance bottlenecks is crucial for advancing the commercial application of ZRFBs.

#### 2.3.1. Ion-Exchange Membranes

Optimizing the properties of the ion-exchange membrane represents a key approach to addressing the challenges confronting ZRFBs. First, enhancing the mechanical strength of the membrane serves as a direct and necessary physical barrier against zinc dendrite puncture [162]. Second, enhancing the membrane’s selectivity toward active species is critical for ensuring long-term cycling stability of the battery, as it not only minimizes side reactions induced by active-species crossover but also homogenizes the distribution of Zn^2+^ to suppress dendrite growth [163]. Furthermore, improving membrane conductivity can promote the uniform deposition of zinc ions, prevent the formation of zinc dendrites, and at the same time help reduce interfacial impedance, thereby enhancing the overall EE of the battery [164].

To address the aforementioned challenges, this section focuses on synergistic design strategies for multifunctional composite membranes, specifically through the precise control of microstructures to optimize three key material properties: mechanical strength, ion selectivity, and ionic conductivity. A foundational strategy involves functionalizing commercial substrates with smart polymer coatings. Pioneering work introduced a porous polyolefin membrane coated with polyethylene glycol (PEG). This coating serves a dual purpose: acting as a Zn^2+^ conductor to homogenize ion flux and forming a passivation layer that inhibits dendritic zinc growth at protrusion tips via exclusion-like interactions. This approach enabled Zn-Br_2_ batteries to operate at an ultra-high current density of 160 mA cm^−2^ with a high areal capacity of 60 mAh cm^−2^ and significantly improved capacity retention [165]. Beyond surface modification, constructing a built-in interfacial electric field offers more precise ionic control. This was exemplified by the in situ growth of CuAl-layered double hydroxide (LDH) nanosheets on a robust polymer blend membrane. The nanosheets generate a surface electrostatic field that guides zincate ions, promoting dense, horizontal zinc deposition. This membrane supported stable cycling for 300 cycles at 200 mA cm^−2^ across a wide areal capacity range (120–260 mAh cm^−2^) [166]. Further advancing this approach, the integration of highly ion-selective covalent organic framework nanosheets onto a similar substrate created a composite membrane that combines mechanical robustness with precise sieving functionality. This design achieved an ultralow area resistance of 0.82 Ω cm^2^, stable operation at 320 mA cm^2^, and dendrite-free cycling over 350 cycles [167]. Pushing the design frontier further involves the strategic alignment of nanofillers within the membrane matrix to create dedicated ion highways. A notable example is the preparation of a hybrid matrix membrane by incorporating zeolitic imidazolate framework nanosheets (ZNSs) into a polybenzimidazole matrix. The high-aspect-ratio nanosheets form straight, aligned ion channels parallel to the membrane surface, eliminating defective pathways. In an alkaline Zn-Fe flow battery, this membrane facilitated stable operation for over 200 cycles at 260 mA cm^−2^ with an average energy efficiency of 82.0%. Molecular dynamics simulations confirmed that these straight channels enable rapid K^+^ transport while effectively blocking Fe(CN)_6_^3−^ crossover, achieving an optimal balance of high conductivity, low permeability, and robust mechanical properties [168].

The number of stable battery cycles can primarily evaluate the growth of zinc dendrites and their impact on membranes. As demonstrated in recent studies (Table 4), advanced modified membranes enable batteries to operate stably for over 100 cycles at current densities exceeding 160 mA cm^−2^ while maintaining an average EE above 80%. In summary, the synergistic optimization of multiple membrane properties—mechanical strength, ion selectivity, and conductivity through microstructural regulation—has proven highly effective in advancing ZRFBs. Future research should prioritize exploring synergistic mechanisms between novel functional nanofillers and polymer matrices. Additionally, integrating theoretical simulations to uncover dendrite suppression mechanisms will accelerate the development of multi-functional composite membranes with high ion flux, superior selectivity, and extended lifespan, thereby driving the practical application of ZRFBs.

#### 2.3.2. Electrolytes

Based on negative electrolyte pH, ZRFBs are primarily categorized into three systems—alkaline, neutral, and acidic—each exhibiting distinct performance characteristics and challenges due to their unique chemical environments [169,170]. The formation of zinc dendrites, HER, other side reactions, passivation, and corrosion of critical battery components constitute the main challenges in electrolyte modification for ZRFBs. To address these issues, strategies include introducing additives such as salts and ionic liquids to regulate zinc ion deposition kinetics and suppress dendrite formation, or incorporating organic chelating agents to coordinate with zinc ions, thereby altering their solvation structure to mitigate interference from side reactions. Meanwhile, each system exhibits distinct performance characteristics and challenges owing to its unique chemical environment [171,172].

The most conventional system is the alkaline system (e.g., Zn(OH)_2_ dissolved in KOH/NaOH). This electrolyte exhibits high ionic conductivity, providing favorable kinetics for zinc deposition and facilitating the formation of smooth, dense zinc deposits. However, in a strong alkaline environment, [Zn(OH)_4_]^2−^ in the electrolyte is prone to decompose into an insulating zinc oxide (ZnO) passivation layer, causing a “tip effect” and triggering dendrites. The strongly alkaline environment exacerbates the HER and accelerates corrosion of battery components [173]. Wang proposed a ligand screening strategy utilizing the stability constant of zinc–ligand complexes (log K) as a quantitative descriptor, identifying nitrilotriacetic acid (NTA, log K = 11.98) at 0.3 M concentration as the optimal chelating agent for alkaline ZRFBs. NTA significantly suppressed parasitic reactions (corrosion current density reduced by 69.9%) while maintaining excellent Zn^2+^ transport properties (diffusion coefficient: 5.77 × 10^−7^ cm^2^ s^−1^). It restructured the Zn^2+^ solvation structure, reduced interfacial water activity, and guided preferential (002) crystal plane growth, enabling dense, dendrite-free zinc deposition. Zinc symmetric cells achieved stable cycling for over 400 h at 80 mA cm^−2^/40 mAh cm^−2^, and Zn-Fe flow batteries delivered 700 cycles (800 h) at 80 mA cm^−2^ with 99% CE [174]. Li introduced 1-ethyl-3-methylimidazolium hexafluorophosphate ([EMIM]PF_6_) as a cost-effective electrolyte additive for alkaline ZRFBs, addressing persistent challenges of HER, corrosion, and passivation. DFT calculations revealed strong preferential adsorption of PF_6_^−^ and EMIM^+^ on zinc surfaces over H_2_O, enabling in situ formation of a robust ZnF_2_-rich solid electrolyte interphase. This protective layer homogenized Zn^2+^ flux, suppressed parasitic reactions, and transformed zinc deposition from diffusion-limited to surface-controlled kinetics. Consequently, Zn//Zn symmetric cells achieved exceptional stability of 311 h at 1 mA cm^−2^/1 mAh cm^−2^, while NiCo-LDH//Zn full batteries delivered 180 mAh g^−1^ at 1 A g^−1^ with 80% capacity retention after 860 cycles [175].

The neutral electrolyte system has developed most rapidly in recent years, such as the electrolyte using zinc sulphate as the main salt [176,177]. During operation of this system, H^+^ generated in the positive electrolyte during charging migrates through the ion-exchange membrane to the negative side, causing the negative electrolyte to gradually shift from neutrality toward acidity. To maintain the electrolyte’s pH stability, a buffer (e.g., acetic acid) is typically added [178]. This system offers a mild environment, resulting in minimal corrosion to key components like battery current collectors and separators, demonstrating excellent material compatibility, while effectively suppressing the HER. However, under this system, a zinc hydroxyl sulfate passivation layer is prone to forming, resulting in uneven deposition and inducing the formation of zinc dendrites [179]. Concurrently, owing to the scarcity of charge carriers, the electrical conductivity of this system is typically low [180]. Zhang employed glycine as a low-cost electrolyte additive to engineer Zn^2+^ solvation structure in neutral ZRFBs. DFT calculations confirmed that the strong coordination bond formed between glycine and Zn^2+^ displaces water molecules from the primary solvation shell, thereby reducing the interfacial water activity. This modification effectively suppressed HER and corrosion, while promoting uniform zinc deposition with a preferential (002) crystal orientation and a dendrite-free morphology. Full Zn-Fe flow battery demonstrated exceptional longevity of 1200 cycles at 30 mA cm^−2^, with EE exceeding 85%. It maintained >70% efficiency at 70 mA cm^−2^, and delivered a peak power density of 160 mW cm^−2^ [181]. Zhang introduced aspartic acid as a universal additive into membrane-free neutral Zn-Mn redox flow batteries to simultaneously address zinc dendrite growth, corrosion, and Mn cathode irreversibility. Asp effectively chelates with Zn^2+^ and Mn^2+^ ions, suppressing side reactions including HER, chlorine evolution, and Mn^3+^ disproportionation. This single-additive strategy enabled the battery to achieve exceptional cycling stability over 300 cycles at a high areal capacity of 10 mAh cm^−2^ with nearly 100% CE. The modified electrolyte demonstrated controlled Mn^2+^ oxidation pathways and uniform zinc deposition morphology [182].

Acidic systems (such as adding H_2_SO_4_ to ZnSO_4_) have received relatively less research at present. This is because metallic zinc has poor tolerance to acids and is prone to corrosion, and the HER is also more severe in an acidic environment. However, this system has a high working voltage, which can further increase the battery capacity. By reducing the zinc deposition overpotential, the system effectively promotes uniform zinc deposition, thereby suppressing dendrite growth to a certain extent [183]. Selecting an appropriate pH environment requires a comprehensive evaluation of the specific battery design objectives, performance targets, and material tolerance limits. While acidic systems in Zn-Fe flow batteries effectively suppress Fe^2+^/Fe^3+^ hydrolysis, replacing strong acids with weak acids is a critical strategy to mitigate acid corrosion on zinc electrodes. Zhang introduced gluconic acid as an additive to substitute for strong acids in acidic Zn-Fe flow batteries. DFT calculations indicated that GA exhibits a strong binding affinity with Fe^3+^, effectively inhibiting both the hydrolysis reaction and transmembrane transfer. This enabled stable operation for 133 cycles (>125 h) under mild acidic conditions (pH = 3–4), extending battery lifetime by 215% compared to conventional batteries requiring pH < 0. The modified system maintained 80.5% EE at 20 mA cm^−2^ while eliminating highly corrosive media, significantly enhancing component durability and reducing environmental impact [184].

Additionally, to overcome the inherent thermodynamic voltage window limitation of aqueous electrolytes, research on alternative systems such as water-in-salt and organic solvent-based electrolytes is intensifying [185,186]. Water-in-salt electrolytes minimize water content through extreme concentration, for inhibiting hydrogen electrolysis reactions and the hydrolysis of active materials. However, significant drawbacks include environmental unsustainability and high operational costs [187]. Organic solvents generally offer a wider electrochemical stability window, holding promise for significantly enhanced energy density and operating voltage. Nevertheless, zinc salts typically exhibit low solubility in non-aqueous media, and such systems commonly face challenges including low ionic conductivity, elevated cost, and safety concerns [188].

Given the increasing complexity and diversity of solvent systems in ZRFBs, composite solvent strategies have gained significant research attention. Zhang proposed the concept of a high-entropy electrolyte. The electrolyte containing multiple solvent components (≥4) with a single solute was achieved by introducing a fixed total volume (20% by volume) of organic solvents (methanol, ethylene glycol, glycerol) into aqueous zinc sulphate solutions. This entropy engineering approach facilitates Zn^2+^ desolvation kinetics with reduced activation energy and creates a cleaner electrode-electrolyte interface with minimal solvent retention during deposition. In practical 100 cm^2^ flow cells operating at ~34% zinc utilization, this electrolyte delivered an exceptional volumetric anode capacity of 1905 mAh cm^−3^, high EE (>84%), and stable cycling beyond 100 cycles [189].

#### 2.3.3. Electrode Materials

Porous carbon materials, represented by CF and GF, are also the most widely used electrode materials in ZRFBs [190]. This is due to their excellent electrical conductivity, good chemical stability, and low cost [191,192]. Meanwhile, porous materials provide abundant deposition sites for zinc, which is conducive to the uniform deposition of zinc and inhibits the formation of zinc dendrites [193]. However, such carbon-based electrode materials commonly suffer from limitations including low electrocatalytic activity, susceptibility to zinc dendrite formation, and inadequate suppression of side reactions. To overcome these challenges, researchers frequently adopt modification strategies by incorporating highly catalytic metallic/non-metallic materials or organic complexes. These approaches aim to tailor the surface active sites of carbon substrates, promote uniform zinc deposition, enhance catalytic activity toward critical electrochemical reactions, suppress parasitic side reactions, and thereby improve the overall EE of the battery.

A pivotal approach involves tailoring the electrode-electrolyte interface to guide zinc deposition morphologically. For instance, a surface passivation strategy was applied to carbon felt electrodes via sputtering a chemically inert alumina layer with a natural compositional gradient. Asymmetric cells using passivated electrodes demonstrated exceptional stability with 800 cycles at 5 mA cm^−2^ and 1600 cycles at 10 mA cm^−2^ without short-circuiting. When integrated into a flow battery, the passivated electrode enabled over 900 stable cycles with minimal capacity fading [194]. Concurrently, the design of bimetallic catalysts introduces bifunctional sites to accelerate complex conversion reactions. A 3D graphene foam electrode modified with an optimized NiFe catalyst exemplifies this, where the bimetallic synergy simultaneously enhances the iodine redox kinetics and suppresses parasitic reactions in neutral Zn-I_2_ systems. This resulted in significantly improved energy efficiency (72.39%) and coulombic efficiency (89%) at 20 mA cm^−2^, underscoring the value of tailored electronic structures for multi-electron transfer processes [195]. Beyond metallic systems, layered double hydroxides have emerged as a highly versatile platform for electrode modification, owing to their tunable interlayer chemistry, excellent ion transport channels, and rich surface hydroxyl groups for enhanced wettability [196]. Researchers have creatively functionalized LDHs to target specific challenges. For example, bismuth-doped MgAl-LDH nanosheet arrays were engineered on carbon felt to serve a dual role: the hierarchical structure increased the active surface area, while bismuth doping created highly active sites that accelerated bromine reaction kinetics and improved species adsorption, leading to a high energy efficiency of ~70% at 160 mA cm^−2^ and stable 1200-cycle operation in a Zn-Br_2_ battery [197]. In a separate approach targeting zincate ion transport, EDTA was intercalated into MgAl-LDH to expand the interlayer spacing. This created rapid diffusion pathways for bulky zincate ions and promoted uniform zinc deposition via a chelation effect, which enabled exceptional stability with a mere 0.004% energy efficiency decay per cycle at 320 mA cm^−2^ in an alkaline system [198].

Table 5 shows that electrode modifications have a smaller impact on ZRFB performance improvement compared to membrane modifications. Some experiments can only operate at low current densities, with long-term cycling EE typically below 70%. This may be attributed to polarization losses caused by zinc dendrites, which reduce VE and subsequently lower EE. In summary, electrode material modifications still hold significant potential for improving ZRFB performance. Future research should focus on multifunctional nanoporous composite materials to optimize interfacial electron structures and ion transport pathways. Additionally, integrating theoretical calculations and AI technologies could enable breakthroughs in novel electrode material discovery.

### 2.4. Organic Redox Flow Batteries

Unlike simply adding organic solvents to the electrolyte, ORFBs represent an energy storage technology that utilizes soluble organic molecules as redox-active materials. These batteries achieve efficient conversion between chemical and electrical energy through electrochemical interface reactions [199]. The core reactions are as follows:

Positive Electrode Reaction: Ox^n_+_^ + n e^−^ ⇌ Red^(n_−k_)+^;

Negative Electrode Reaction: Red^(m−l)+^ − m e^−^ ⇌ Ox^m+^;

Overall reaction: Ox^n_+_^ + Red^(m−l)+^ ⇌ Ox^m+^ + Red^(n_−k_)+^.

In these equations, Ox and Red represent the oxidized and reduced forms of the organic molecules, respectively.

Compared to traditional flow battery systems based on inorganic ions like vanadium or zinc, research on organic flow batteries started later and has followed a more complex development path. In the early 21st century, Smith et al. first published research on quinone redox reactions, followed by a series of studies on ORFBs [200,201]. However, organic molecules generally suffer from limitations like poor chemical stability and short cycle life. These bottlenecks made it difficult to meet practical application requirements, causing research progress to remain slow for a long period [202,203].

The introduction of sulfonic acid groups into the anthraquinone molecule by Aziz and co-workers significantly enhances its solubility in aqueous electrolytes and improves chemical stability, thereby laying a crucial foundation for the practical application of organic flow batteries [204]. Subsequently, Hollas et al. introduced phenazine compounds into the system, which exhibited high redox potentials and excellent reversibility, further advancing research on organic active substances [205]. With the deepening of molecular design concepts, new active material systems emerged. These include polysulfide/quinone hybrid systems and organic-inorganic hybrid systems. These developments have promoted the diversification of organic flow battery technology.

Currently, organic flow batteries have developed several representative technical routes. These include quinone-based flow batteries (using anthraquinone, naphthoquinone, and their derivatives), phenazine-based flow batteries (centered on phenazine and phenothiazine), thiophene-based flow batteries (such as thiophene and its derivatives), and (2,2,6,6-tetramethylpiperidin-1-yl)oxyl flow batteries [206,207,208,209,210,211,212]. However, this technology is still in a critical transition phase from the laboratory to industrialization. It faces multiple challenges, including relatively low energy density, insufficient long-term material stability, high synthesis costs for high-performance organic molecules, and certain safety risks.

## 3. Conclusions

Recent advances in RFB technologies have demonstrated significant progress toward practical large-scale energy storage applications. Systematic research on electrode modification, ion-exchange membranes, and electrolyte engineering has substantially enhanced the performance of all mainstream RFB platforms. For VRFBs, innovations in mixed-matrix membranes and catalytic electrode modifications have markedly improved EE and cycle life, while diverse electrolyte stabilizers have largely resolved vanadium precipitation issues under high-concentration electrolyte conditions. ICFBs have benefited from bismuth- and tin-based catalysts that effectively overcome sluggish Cr^3+^/Cr^2+^ reaction kinetics and suppress the HER, bringing these cost-effective systems closer to commercialization. Meanwhile, ZRFBs have achieved breakthrough progress through innovative strategies, including high-strength composite modified membranes, diverse electrolyte systems, and modified electrode.

Despite these notable advancements, critical challenges remain before widespread commercial deployment. The fundamental trade-offs among energy density, efficiency, and cost require further optimization. VRFBs continue to face economic viability concerns due to vanadium price volatility; ICFBs require improved long-term cycling stability at lower operating temperatures; and ZRFBs demand more robust solutions for managing zinc dendrite formation under high-utilization conditions. In addition, ORFB systems still face many challenges on the path of diversified exploration today.

As renewable energy penetration increases globally, RFB technologies are uniquely positioned to provide the grid stability and long-duration storage required for a sustainable energy future. Through continued innovation in materials science and system engineering, coupled with supportive policy frameworks, flow batteries can overcome current limitations and achieve their full potential as reliable, cost-effective solutions for grid-scale energy storage, ultimately contributing to global decarbonization efforts while enhancing energy security.

## Figures and Tables

**Table 1:** Summary of modified membrane data for VRFBs.

Ref.	Pristine Membrane	Modifying Materials	Current Density	EE		Cycle Life	
				Before Modification	After Modification	Before Modification	After Modification
[73]	PBI	Phosphorylated COF	120 mA cm^−2^	77.9%	85.5%	500 cycles	1000 cycles
[74]	PVDF-co-HFP	Acid-Functionalized Graphene Oxide	100 mA cm^−2^	70.84%	84.21%	904 min	1290 min
[75]	SPEEK	SnO_2_	100 mA cm^−2^	84.0%	86.47%	/	500 cycles
[76]	SPEEK	Zwitterionic polymer-modified MOFs	100 mA cm^−2^	75.0%	87.1%	2292 min	7620 min

**Table 2:** Summary of modified electrode data for VRFBs.

Ref.	Pristine Electrode	Modifying Materials	Current Density	EE		Stability	
				Before Modification	After Modification	Cycle Life	EE Retention Rate
[96]	CF	MXene-SnO_2_	100 mA cm^−2^	80%	85%	500	≈100%
[97]	GF	Nitrogen-doped Vertical Graphene	200 mA cm^−2^	65.9%	87.1%	1500	95%
[98]	GF	Bi, N	220 mA cm^−2^	74.32%	80.26%	600	98.2%

**Table 3:** Summary of modified electrode data for ICFBs.

Ref.	Pristine Electrode	Modifying Materials	Operating Temperature	EE (at 120 mA cm^−2^)		Stability	
				Before Modification	After Modification	Cycle Life	EE Retention Rate
[141]	GF	Bi, Fe, In, Sn, and Ti	40 °C	60%	87.6%	400	83%
[142]	CF	Bi-MOF	65 °C	79.67%	81.69%	100	/
[143]	GF	Sn	65 °C	71.65	85.74%	100	91%
[144]	GF	Sn/SnO_x_	40 °C	75.3%	86.4%	100	/

**Table 4:** Summary of modified membrane data for ZRFBs.

Ref.	Pristine Membrane	Modifying Materials	Current Density	Cycle Life	Average EE
[165]	Daramic membrane	PEG	160 mA cm^−2^	100	/
[166]	PES-SPEEK	CuAl-LDH	200 mA cm^−2^	300	84.8%
[167]	PES-SPEEK	COF	320 mA cm^−2^	200	81.3%
[168]	PBI	ZNSs	260 mA cm^−2^	200	82%

**Table 5:** Summary of modified electrode data for ZRFBs.

Ref.	Pristine Electrode	Modifying Materials	Current Density	Cycle Life	Average EE
[194]	CF	Al_2_O_3_	5 mA cm^−2^	800	/
[195]	GF	Ni, Fe	20 mA cm^−2^	40	67%
[197]	CF	Bi-LDH	160 mA cm^−2^	1200	68.77%
[198]	CF	EDTA@LDH	320 mA cm^−2^	300	53.8%

## Data Availability

Research data are available upon reasonable request by contacting the corresponding author.

## Abbreviations

The following abbreviations are used in this manuscript:

VRFBs	vanadium redox flow batteries
ICFBs	iron-chromium redox flow batteries
ZRFBs	zinc-based redox flow batteries
ORFBs	organic redox flow batteries
EE	energy efficiency
CE	coulombic efficiency
VE	voltage efficiency
SPEEK	sulfonated poly(ether ether ketone)
PBI	polybenzimidazole
PEG	polyethylene glycol
COF	covalent organic framework
MOF	metal-organic framework
LDH	layered double hydroxide
ZNFs	zeolite imidazole framework nanosheets
MWCNT	multi-walled carbon nanotube
CF	carbon felt
GF	graphite felt
CP	carbon paper
CC	carbon cloth
HER	hydrogen evolution reaction
PDTA	1,3-propanediaminetetraacetate
